# Estrogen receptor 1 expression and methylation of *Esr1* promoter in mouse fetal prostate mesenchymal cells induced by gestational exposure to bisphenol A or ethinylestradiol

**DOI:** 10.1093/eep/dvz012

**Published:** 2019-08-22

**Authors:** Ramji K Bhandari, Julia A Taylor, Jennifer Sommerfeld-Sager, Donald E Tillitt, William A Ricke, Frederick S vom Saal

**Affiliations:** 1 Department of Biology, University of North Carolina at Greensboro, Greensboro, NC, USA; 2 Division of Biological Sciences, University of Missouri, Columbia, MO, USA; 3 United States Geological Survey Columbia Environmental Research Center, Columbia, MO, USA; 4 Department of Urology, Molecular Environmental Toxicology Program, George M. O’Brien Center of Research Excellence, University of Wisconsin, Madison, WI, USA

**Keywords:** prostate mesenchymal cells, bisphenol A, ethinylestradiol, DOHaD, hormone action, plastic, estrogen receptor, fetal exposure, DNA methylation, epigenetics

## Abstract

Fetal/neonatal environmental estrogen exposures alter developmental programing of the prostate gland causing onset of diseases later in life. We have previously shown *in vitro* that exposures to 17β-estradiol (E2) and the endocrine disrupting chemical bisphenol A, at concentrations relevant to human exposure, cause an elevation of estrogen receptor α (*Esr1*) mRNA in primary cultures of fetal mouse prostate mesenchymal cells; a similar result was observed in the fetal rat urogenital sinus. Effects of these chemicals on prostate mesenchyme *in vivo* are not well understood. Here we show effects in mice of fetal exposure to the estrogenic drug in mixed oral contraceptives, 17α-ethinylestradiol (EE2), at a concentration of EE2 encountered by human embryos/fetuses whose mothers become pregnant while on EE2-containing oral contraceptives, or bisphenol A at a concentration relevant to exposures observed in human fetuses *in vivo*. Expression of *Esr1* was elevated by bisphenol A or EE2 exposures, which decreased the global expression of DNA methyltransferase 3A (*Dnmt3a*), while methylation of *Esr1* promoter was significantly increased. These results show that exposures to the environmental estrogen bisphenol A and drug EE2 cause transcriptional and epigenetic alterations to expression of estrogen receptors in developing prostate mesenchyme *in vivo*.

## Introduction

Bisphenol A (BPA) is a high-production-volume chemical (∼20 billion pounds/year) used in a wide variety of consumer products: plasticizer in polyvinyl chloride and other plastics, component of plastic linings of food and beverage containers, composites used in dentistry, and as a developer in thermal paper, to name just a few products [1]. It is impossible to know all sources of BPA exposure; however, data from the US National Health and Nutrition Evaluation Survey, as well as other biomonitoring studies, indicate that exposure to BPA is ubiquitous and from multiple sources [2–4]. BPA has been found in food, beverages, air, water, and soil [5–9]. BPA is capable of affecting cell signaling mechanisms involving estrogen, androgen, aryl hydrocarbon, and thyroid hormone receptors and has been found to cause a multitude of adverse health outcomes in model organisms, such as metabolic syndrome, reproductive, neurobehavioral, immune defects, and cancer [8]. Numerous epidemiological studies have reported associations between BPA and adverse health effects in humans [10].

Fetal urogenital system development is regulated by gonadal hormones that regulate autocrine and paracrine factors [11]. In mice, prostate differentiation begins with the development of glandular buds from the proximal urogenital sinus (UGS) at gestational day 17 under the control of 5α-dihydrotestosterone. Mesenchymal cells in the differentiating prostate express androgen receptor (*Ar*) that has a higher affinity for 5α-dihydrotestosterone relative to testosterone. Testosterone does not circulate at high enough concentrations to stimulate gland genesis, thus requiring the expression of 5α-reductase in mesenchymal cells for gland genesis to occur [12]. At the same time, estrogen receptor alpha (*Esr1*) is also expressed in the mesenchyme, which suggested a role for endogenous estrogen in modulating prostate development [13]; this was subsequently confirmed, although the effects of estradiol and other estrogens (stimulatory vs. inhibitory) on prostate gland genesis and subsequent function are highly dependent on dose. Specifically, our previous studies show that increasing exposure to estrogens within a physiologic range (referred to as “low doses”) results in a permanent increase in the size and androgen responsiveness of the prostate [14]. In contrast, fetal exposure to estrogenic chemicals within the high-dose, pharmacologic range has the opposite effect of inhibiting prostate development [14, 15]. In contrast, UGS epithelial cells do not respond directly to androgen, but estrogens within a physiological dose range stimulate their proliferation via activation of Esr1 in mesenchyme [15, 16]. It has been known for some time that mesenchyme regulates epithelial proliferation in the developing UGS [11, 17, 18].

The possibility that BPA could alter epigenetic mechanisms that are predicted to be the regulators of gene expression was examined in a mouse model in which a retrotransposon inserted upstream of the transcription start site of the *Agouti* gene. Expression of this gene led to a yellow coat color, as well as obesity, diabetes, and numerous tumors, which were related to differential methylation in the cryptic promoter region of the retrotransposon [19]. Using this model, Dolinoy *et al.* showed differential methylation of the retrotransposon promoter by BPA as opposed to the phytoestrogen genistein. Exposure to BPA resulted in hypomethylation of the promoter while genistein exposure had the opposite effect of hypermethylation of the promoter. This “proof of principle” experimental study revealed that developmental exposure to BPA could alter the epigenome, resulting in a dramatic effect on adult phenotype.

Maternal exposure to BPA or ethinylestradiol (EE2) causes an increase in the number of developing prostatic glands in male fetuses, with the dose of BPA required being ∼100-fold > EE2 [20], although both the BPA and EE2 doses were within the range of human exposure from BPA-containing products and oral contraceptives, respectively [3, 21–23]. Studies by us and others suggest that environmental estrogenic chemicals affect programing of estrogen responsiveness within the fetal prostate, resulting in permanent changes at the molecular and cellular levels [24]. Exposure to estradiol-17β increased expression of *Esr1* and *Ar* in mouse prostate mesenchymal cells in primary culture [25], and BPA caused a similar increase [18]. However, it has not been determined whether the *in vitro* findings showing effects on *Esr1* expression from studies of fetal mesenchyme in primary culture also occur as a result of administration of BPA or estrogenic drugs such as EE2 *in vivo*. In the present study, we examined changes in *Esr1* transcripts and DNA methylation on *Esr1* promoter in CD-1 mouse fetal prostate mesenchymal cells caused by *in utero* exposure to low doses, within the range of human exposure, of BPA and EE2.

## Results

### BPA Effects on Estrogen Receptor and Aromatase Expression

**Figure 1:**
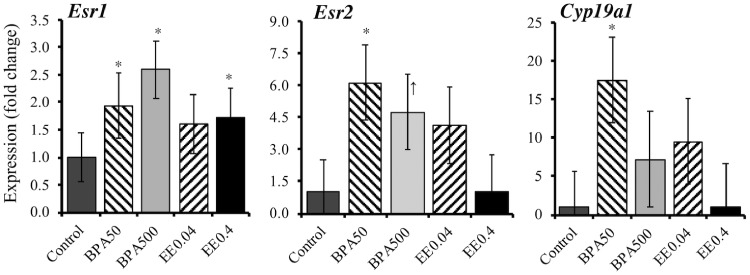
increase in expression of *Esr1*, *Esr2*, and *Cyp19a* relative to controls (set at 1) in UGS mesenchyme from male fetuses on GD 17 after exposure to BPA (50 or 500 µg/kg/day) or ethinylestradiol (EE2) at doses of 0.04 and 4 µg/kg/day. Total of 8 biological replicates were used for control and 5 biological replicates each for rest of the treatments. *P < 0.05 and ^↑^*P* < 0.1 > 0.05

We examined the effects of *in utero* BPA exposure on expression of genes encoding estrogen receptors *Esr1 and Esr2* (Fig. 1). Both doses (50 and 500 µg/kg/day) of BPA significantly (*P* < 0.05) increased expression of *Esr1*, whereas only the higher (0.4 µg/kg/day) dose of EE2 significantly (*P* < 0.05) elevated *Esr1* expression (Fig. 1). For *Esr2*, the lowest dose of BPA significantly (*P* < 0.05) increased expression, while the higher BPA dose tended (*P* = 0.08) to increase expression. However, neither dose of EE2 significantly increased *Esr2* expression (*P* > 0.1; Fig. 1). Expression of *Cyp19a* was at the lowest level of detection in control cells. The lower dose of BPA significantly increased *Cyp19a* expression (*P* < 0.05), while the lower (0.04 µg/kg/day) dose of EE2 tended to induce *Cyp19a* expression (*P* = 0.07). The results for both BPA and EE2 suggested a greater stimulatory effect of *Cyp19a* at lower doses relative to higher doses of each chemical.

### BPA Effects on DNA Methyltransferase Gene Expression

**Figure 2:**
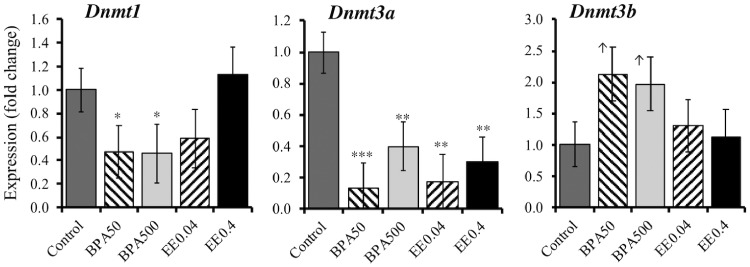
change in expression of *Dnmt1*, *Dnmt3a*, and *Dnmt3b* relative to controls (set at 1) in UGS mesenchyme from male fetuses on GD 17 after exposure to BPA (50 or 500 µg/kg/day) or ethinylestradiol (EE2) at doses of 0.04 and 4 µg/kg/day. Total of 8 biological replicates were used for control and 5 biological replicates each for rest of the treatments. *P < 0.05, **P < 0.01, ***P < 0.001, and ^↑^*P* < 0.1 > 0.05

The mRNA for maintenance methyltransferase, *Dnmt1*, was significantly decreased by both BPA doses (*P* < 0.05), but not by either dose of EE2 (*P* > 0.1) (Fig. 2). *De novo* methyl transferase, *Dnmt3a*, was significantly decreased by both doses of BPA and EE2 (*P* < 0.01). *Dnmt3b* expression tended to increase in response to the lower dose of BPA (*P* = 0.055) and the higher dose of BPA (*P* = 0.096), but was not significantly changed by either dose of EE2.

### In Silico Analysis of Esr1 and Esr2 Promoters

**Figure 3:**
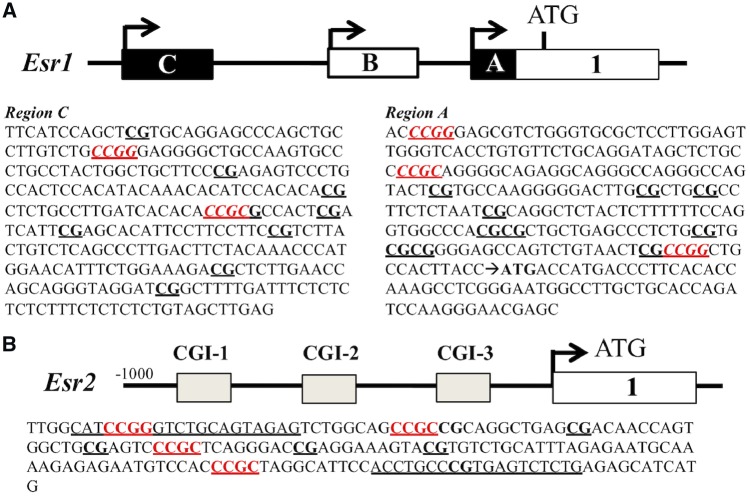
(**A**) the sequence of mouse promoter Esr1 Exon 1C (GRCm38/mm10, Dec 2011 Assembly). The sequence was deducted from mouse chromosome 4,709,981–4,710,280 from Ensembl genome database (100% match). Sequence for promoter Esr1 exon 1A was deducted from mouse chromosome 10: 4,712,236–4,712,507 from Ensembl genome database (100% match). (**B**) sequence of Esr2 promoter was deducted from mouse chromosome 12: 76,176,940–76,177,091 at Ensembl genome database (100% match). Red underlined nucleotides represent MSRE cut site (C**CG**G for HpaII and C**CG**C for AciI) in both sequences. Internal control sequence was obtained from the chromosome 10: 4,711,011–4,713,710 and contained no CpG site

The mouse *Esr1* exon A promoter was selected for our studies based on findings of Kundakovic *et al*. [26]. We also conducted *in silico* analysis for the structure of the 500-bp promoter region to determine *Esr1* and *Esr2* core promoter CpG content. The *Esr1* exon 1A region contains a CpG island of 118 bp and two restriction cleavage sites for HpaII (CCGG) and a site for AciI (CCGC). The *Esr1* exon 1C region contains a CpG island of 108 bp as well as a site for HpaII and one for AciI (Fig. 3). The *Esr2* promoter contains 3 CpG islands. The CGI #3 has 12 CpGs, a HpaII site and 3 AciI sites within the 500-bp upstream of the ATG site (Fig. 3).

### Methylation of Esr1 and Esr2 Promoters and Global DNA Methylation

**Figure 4:**
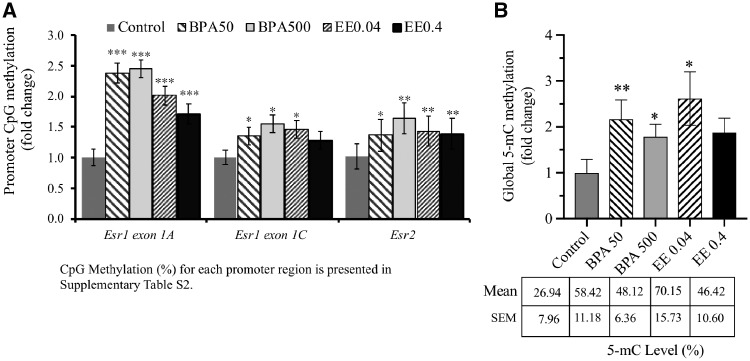
(**A**) methylation of CpGs in *Esr1 Exon 1A*, *1C*, and *Esr2* promoters relative to no CpG controls (set at 1) in UGS mesenchyme from male fetuses on GD 17 after exposure to BPA (50 or 500 µg/kg/day) or ethinylestradiol (EE2) at doses of 0.04 and 4 µg/kg/day. Methylation was accounted for cytosine methylation of C**CG**G or C**CG**C of two restriction sites. There were 3 CpG sites in Esr1 Exon 1A, 4 in Exon 1C, and 4 in Esr2 core promoter. Percent methylation in each treatment group is being presented in Supplementary Table S2. Total of 7 biological replicates were used for control and 5 biological replicates each for rest of the treatments. *P < 0.05, **P < 0.01, ***P < 0.001, and ^↑^*P* < 0.1 > 0.05. (**B**) global DNA methylation on UGS mesenchymal cells in response to gestational exposure to BPA and EE2 in mice on gestational day 17. Global DNA methylation is presented as fold-change difference against control. Percent methylation per treatment is given in a table underneath. *Statistical significance at the level of *P* < 0.05

Methylation of select CpGs in the CpG island of *Esr1* exon 1A was significantly increased (*P* < 0.001) by *in utero* exposure to BPA or EE2. Methylation of select CpGs in the CpG island of *Esr1* exon 1C was also significantly increased (*P* < 0.05) by *in utero* exposure to either dose of BPA and the lower dose of EE2, while the higher dose of EE2 tended to increase CpG methylation (*P* = 0.08) (Fig. 4A). The methylation of *Esr2* promoter CpG island was significantly increased with both doses of BPA and EE2 (*P* < 0.05). The average methylation of genome DNA in isolated mesenchymal cells was significantly increased by both BPA treatments and low EE2 treatment (Fig. 4B).

## Discussion

The present study demonstrates that *in utero* exposure of developing mouse embryos [gestation day (GD) 11–17] to the 50-µg/kg/day BPA dose (estimated by the EPA and FDA to be the safe daily oral exposure dose for BPA) altered expression of *Esr1*, *Esr2*, aromatase, and DNA methyltransferase genes in mesenchymal cells in the developing UGS of fetal male mice (Figs 1 and 2). EE2 at doses equivalent to those in mixed oral contraceptives (0.4 µg/kg/day), and a 10-fold lower dose (0.04 µg/kg/day), caused many, but not all, of these same effects. While this could suggest that some effects of BPA may not be due only to its known estrogenic activity [8], it is well recognized that effects of different estrogenic chemicals on gene expression are dose dependent, and with only two doses per chemical, it is not possible to conclude that BPA and EE2 are causing effects through different response mechanisms [27]. Additionally, both BPA and EE2 elevated DNA methylation levels (5-mC) on *Esr1 exon 1A* and *1C* (Fig. 4A). Global DNA methylation levels also showed a similar pattern to *Esr1* promoter methylation (Fig. 4B), indicating that BPA exposure caused hypermethylation of not only estrogen receptor genes but also many other genes in the genome of developing fetal mesenchymal cells, which ultimately regulate development of the prostate gland [11, 12].

These *in vivo* findings are consistent with our previous *in vitro* studies revealing BPA-induced alterations in a suite of genes, including *Esr1*, involved in prostate development [18, 25]. This study also provides new insights into the epigenetic basis for BPA disruption of normal development of the prostate. The data in the present study were collected from an isolated UGS mesenchymal cells from GD 17 male mouse fetuses, and gene expression and DNA methylation were measured in the same cell population. It is important to use a homogenous cell population for epigenetic studies, since non-target cells in the tissue can dissociate epigenetic information. For example, the same gene that is expressed in the target cells may be suppressed in non-target cells by epigenetic repressive mechanisms. In our previous studies, BPA exposure altered *Esr1* expression in prostate mesenchyme in a non-monotonic manner *in vitro*, with low but not high doses stimulating expression [18, 25], but whether BPA could induce similar changes in *Esr1* expression *in vivo* was not clear.

Our results suggest that the oral doses of BPA that we used in this study, which produce levels of unconjugated (bioactive) BPA (0.01–0.1 ng/ml) in fetal mouse serum [28], which are equivalent to those found in numerous human biomonitoring studies [4, 29, 30], increase *Esr1* and *Esr2* expression, as well as expression of aromatase; the increase in aromatase activity has previously been reported in fetal mouse UGS mesenchyme [31] and other tissues in cell culture [32]. BPA thus not only can itself interact with estrogen receptors but increase the intracellular levels of estradiol by stimulating an increase in aromatase activity in prostate mesenchyme.

Estrogens modulate prostate growth and differentiation in mammals through *Esr1*. During the prenatal and neonatal period of differentiation, *Esr1* is expressed in UGS mesenchyme [20], but not UGS epithelium [33]. Since cytokines secreted by mesenchyme regulate epithelial differentiation in the UGS, this supports predictions that both endogenous and exogenous estrogen signaling play a role in the development of prostate morphology in mice [14, 16, 20]. In addition to studies in mice, in rats it was shown that BPA was able to alter estrogen signaling in early stages of UGS organogenesis, and these changes were linked to adverse health outcomes later in life. Specifically, a transient developmental exposure of rats to low, environmentally relevant doses of BPA was found to increase prostate gland susceptibility to adult-onset precancerous lesions and hormonal carcinogenesis [24, 34]. *Esr1* is expressed in mesenchymal cells during the early postnatal period, whereas *Esr2* expression in prostatic epithelium peaks during postnatal week 4 after completion of branching morphogenesis of prostate ducts; ESR1, but not ESR2, has been found to modulate estrogen-mediated prostrate differentiation in mice [16].

Our previous studies have shown that exposure of male mouse fetuses to slightly elevated endogenous and exogenous estrogen levels results in gross malformations in the UGS, an increase in the number of prostatic ducts, and a permanent enlargement of the prostate associated with elevated AR levels in adulthood [14, 20]. These effects occur due to a number of factors: it has been shown that BPA increases the production of estradiol by aromatase in the developing prostate mesenchyme [31], and we report here that BPA increased expression of the aromatase gene (*CYP19a*) as well as increased *Esr1* expression in the same mesenchymal cell population. Taken together, these findings suggest that a low dose of BPA can induce estrogenic effects in developing prostate mesenchyme by altering the epigenetic programing of Esr1 and Esr2, which could alter the proliferative vs. anti-proliferative balance of these receptors, leading to adult prostate pathology [16]. To support the concept of ERα playing a role in prostate pathogenesis in adults, loss of ERα function experiments has prevented the development of prostatic carcinogenesis and benign prostate hyperplasia in mouse models [35]. In addition, since BPA can serve as an agonist for estrogen receptors, an increase in aromatase, induced by BPA would result in elevated estradiol levels in estrogen-target mesenchymal cells that express Esr1 during fetal life, resulting in a “double hit” of additional estrogen, both exogenous [BPA and endogenous (estradiol)]. Additional research will be required to examine whether the epigenetic perturbations we observed during prostate development lead to adult-onset prostate disease. Considerable evidence exists that in rodents, a “two-hit” model in which both developmental and then adult estrogen treatment is required to observe prostate pathology in adulthood [34].

To investigate involvement of epigenetic mechanisms, specifically, DNA methylation, during fetal exposure of mesenchymal cells to BPA, we measured expression levels of *Dnmt* genes in the isolated mesenchymal cells. The expression level of *Dnmt3a* was significantly decreased by BPA and EE2 exposure (Fig. 2), suggesting that BPA exposure should have suppressed *de novo* methylation during early organogenesis in the UGS in male fetuses. The expression pattern of *Dnmt3b* in response to BPA and EE2 treatments (Fig. 2) showed similar patterns to *Esr* expression and promoter methylation profile in these cells (Fig. 1), suggesting Dnmt3b’s possible role in prostate differentiation. Studies suggest that Dnmt3b influences genomic patterns of methylation *in vivo* by interacting with Dnmt3a and Dnmt3L and fulfilling their epigenetic roles [36]. *Dnmt1* expression is variable in developing prostate. In C57 mice, *Dnmt1* expression begins to localize to basal epithelium and prostatic buds by GD 17.5, while *Dnmt3a* and *Dnmt3b* localize to prostatic buds by postnatal day 5. Interestingly, Dnmt1 exclusively localizes in a spotty pattern to periurethral mesenchyme (lamina propria and submucosa) of the male lower urinary tract, suggesting that prostate development in the male involves DNA methylation [37]. Our present study suggests that BPA perturbs early programing of *Dnmt* expression in the UGS mesenchyme, which may or may not maintain this pattern until adulthood. Human prostate stem-progenitor cells are direct BPA targets, and developmental exposure to BPA at low doses increases hormone-dependent cancer risk in human prostate epithelium [38]. In addition, *Dnmt1* expression increases in human prostate cells undergoing carcinogenesis [39].

The CpG methylation on the select region of *Esr1* exon 1A, *Esr1* exon 1C, and *Esr2* promoters was increased by *in utero* BPA and EE2 exposure (Fig. 4A). Interestingly, a similar pattern was observed for global methylation of the UGS mesenchyme genome (Fig. 4B), indicating that BPA and EE2 induce hypermethylation in the developing prostate. Involvement of DNA methylation in early prostate development and regulation of *Ar* has been demonstrated by culturing developing prostate with an inhibitor of DNA methylation, 5-aza-cytidine [40].

The association of DNA methylation with *Esr1* and *Esr2* expression is not sufficient to explain the role of DNA methylation in transcription of genes we examined in developing UGS mesenchymal cells. Many other mechanisms are likely involved in the regulation of these genes, in addition to the select CpG islands that we examined. Technically, the presented DNA methylation assay involved the use of the methylation-sensitive restriction enzymes (MSREs) (e.g. AciI, HpaII) that cut only unmethylated DNA, but not methylated DNA [41]. The use of quantitative polymerase chain reaction (qPCR)-based detection enabled a reliable and simple detection of DNA methylation targeting native DNA sequences. This method is superior to the presumed gold standard method that involves bisulfite conversion of genomic DNA, PCR, cloning, and sequencing of only a few clones (representing <1% of total cells in the sample), as this method takes into account the total number of fragments that are not cleaved by MSREs.

The initial dogma in epigenetics was that tissue-specific genes are methylated in the tissues in which they are not expressed and remain unmethylated in the tissues in which they are expressed [42], and in many cases DNA methylation is, in fact, negatively correlated with target gene expression. In contrast, in the present study, gene expression was elevated despite an increase in DNA methylation of CpGs in the restriction enzyme cleavage sites. This pattern of a positive relationship between gene expression and CpG methylation is actually not uncommon. Studies by Wan *et al.* [43] suggest that a significant portion of tissue-specific differentially methylation regions are positively correlated with gene expression, and these positive tissue-specific differentially methylated regions are more enriched in the promoter regions. The target genes for these positive tissue-specific differentially methylation regions are enriched with distinct sets of DNA sequence motifs for negative regulators, such as transcriptional repressors, suggesting a novel mode of indirect DNA methylation inhibition of expression through transcriptional repressors. A study by Nakamura *et al*. [44] suggests that irrespective of the DNA methylation status of the promoter, repressive histone marks are still able to alter gene expression. In BALB/c mice, Kundakovic *et al*. [26] found that BPA exposure induced a tissue-specific methylation pattern in the brain, mainly increasing DNA methylation in the prefrontal cortex and decreasing methylation in the hypothalamus, suggesting that tissue-specific transcriptional activation of a gene is controlled by several epigenetic and transcriptional regulators.

In summary, the present study demonstrates a distinct global DNA methylation profile on the UGS mesenchyme genome, unique pattern of Esr promoter methylation, and expression pattern of Dnmt genes in the isolated mesenchymal cells examined at GD 17 after maternal exposure to both low doses of BPA and EE2. Our data thus suggest involvement of epigenetic mechanisms in prostate development and their alteration by exposure to BPA and EE2. Future studies are directed at pinpointing epigenetic changes in prostate mesenchyme by next generation sequencing of the epigenome and step-by-step tracing of epigenetic and gene expression profiles in parallel with the later onset of prostate disease phenotype.

## Methods

### Animal Maintenance and Tissue Collection

CD-1 mice purchased from Charles River Laboratories (Wilmington, MA) were housed in a facility accredited by the Association for Assessment and Accreditation of Laboratory Animal Care at the University of Missouri. All mice were kept in standard polypropylene cages with corncob bedding and glass water bottles filled with BPA-free water purified by reverse-osmosis and carbon filtration. Room temperature was maintained at 25 ± 2°C under a 12 h:12 h light:dark cycle. Female mice were fed with Purina chow (5008) throughout the experiment and had been maintained on Purina 5001 chow prior to mating. Animal procedures were approved by the University of Missouri Animal Care and Use Committee and conformed to the NIH *Guide for the care and Use of Laboratory Animals*. Between five to eight timed-pregnant females were used for each treatment.

### Treatment of Animals and Tissue Collection

Beginning on GD 11 (conception was GD 0), pregnant mice were fed (via micropipetter) BPA one time per day in tocopherol-stripped corn oil (MP Biomedical, Santa Ana, CA); the volume administered (∼30 µl) was adjusted based on individual body weights to contain doses of 50 and 500 µg/kg/day (each dose was obtained from a different stock solution), using methods described previously [28]; the 50 µg/kg/day dose is the EPA reference (safe daily exposure) dose, while the 500 µg/kg/day dose is below the EPA’s estimated no effect dose [45]. EE2 doses were adjusted according to the studies published previously by us and others [18, 25, 46]. Briefly, animals were weighed daily and fed just vehicle (controls) or a volume of BPA solution adjusted to maintain a constant dose/kg body weight. EE2 was administered in the same manner at 0.04 and 0.4 µg/kg/day, the latter dose being similar to the dose of EE2 in mixed oral contraceptives.

Treated timed-pregnant females were euthanized on GD 17 by CO_2_ asphyxiation, fetuses were collected, and sex was determined by examining for the presence of testes or ovaries. Only male fetuses were used, and the UGS was collected using methods previously described in detail [18, 25]. Briefly, cells from the UGS tissue were dispersed by digestion with collagenase type I. Epithelial and mesenchymal cells in the suspension were separated by gravity, since the epithelial cells settle, and the mesenchymal cells remain suspended. The collected mesenchymal cells were washed in DMEM and centrifuged. The medium was discarded, and the cell pellet was snap frozen in liquid nitrogen and stored at −80°C until analysis. This method yields a homogeneous population of prostate mesenchymal cells. In prior studies, we had characterized the cell-type composition by immunofluorescent staining of cytokeratins with mouse anti-pan-cytokeratin clone PCK-26 fluorescein isothiocyanate conjugate (Sigma), and co-staining of the mesenchymal cell marker vimentin with goat anti-vimentin (Sigma) and rabbit anti-goat Cy3 conjugate (Sigma) [18, 25].

### RNA and DNA Collection and Real-Time Quantitative PCR

Cell pellets were suspended and homogenized in lysis buffer (Qiagen RNA/DNA Combo kit; Qiagen, California, USA), and DNA and RNA were extracted according to the manufacturer’s instructions. RNA was DNAase-treated, and cDNAs were synthesized using 2 µg RNA with 12–18 mer oligo dT primers and MMLV reverse transcriptase (Promega, WI, USA). cDNAs were stored at −20°C until analysis. DNA from the same cells was extracted according to the manufacturer’s manual (Qiagen) and stored at −20°C until analysis. Both RNA and DNA were quantified by nanodrop 1000 (Thermo Fisher, USA). Genes that were used for qPCR analysis were estrogen receptors (*Esr1*, *Esr2*), DNA methyltransferases (*Dnmt1*, *Dnmt3a*, and *Dnmt3b*), and aromatase (*Cyp19a*). *Gapdh* was used as an internal control for qPCR assay. Primers were designed from exon–intron boundary (Supplementary Table S1) and tested for optimum Tm. Real-time qPCR was performed using SYBR Green master mix and target primers on an ABI qPCR instrument 7000. The Real-time qPCR assays for each sample were carried out in duplicate. The relative concentrations of specific mRNAs in each sample were normalized to the internal control *Gapdh*, as described. Fold-change differences in expression were calculated by the 2^−^^ΔΔCt^ method.

### Methylation-Sensitive Restriction Enzyme qPCR

We examined DNA methylation of the *Esr1* and *Esr2* gene promoters in the same cells in which their gene expression was measured by using the MSRE-qPCR method. Prior to MSRE-qPCR assay with experimental samples, we validated the assay with known positive and negative control DNA. The qPCR amplification efficiency was between 95% and 99%, suggesting that the assay is suitable for measuring DNA methylation in select CpG sites (data not shown). Sample DNA (1 µg) was digested with MSRE HpaII (CCGG) and AciI (CCGC) at 37°C for 1 h, and enzyme was deactivated at 80°C for 20 min. *Esr1 exon 1A*, *Esr1 exon 1C*, and *Esr2* promoters were selected and primers designed (Fig. 4A). For qPCR, primers were designed from target promoter flanking CpG sites of interest or CpG island-containing sites for restriction cleavage. The CpG sites and restriction digestion sites for HpaII and AciI are shown in Supplementary Fig. S1. Internal control primers for normalization were designed from the genomic region without restriction cleavage sites and CpGs (see Supplementary Information for primer sequences, Supplementary Fig. S1D). A total of 8 ng DNA and 3 well replicates were used for qPCR of each sample. DNA methylation differences were calculated using the 2^−^^ΔΔCt^ method and expressed as fold difference relative to the control, which is 1.

### Global DNA Methylation Assay

Global changes in methylation of UGS mesenchyme genomic DNA were measured by commercial kit from Zymo Research (California, USA) and validated using internal standards (positive and negative controls) and a standard curve. To quantify global changes in DNA methylation, 100 ng DNA was denatured and used for 5-mC global DNA methylation ELISA (Zymo Research) according to manufacturer’s instructions. The standard curve was generated, and DNA methylation readings were extrapolated using equation $\left\{\%5-\text{mC}=e^{\left[(\text{Absorbance}-\;y-\text{intercept})/\text{Slope}\right]}\right\}$ provided by the manufacturers. To find the fold-change difference in global methylation of genomic DNA in UGS mesenchymal cells, experimental values were divided by the average of control values (Fig. 4B).

### Statistics

We used ANOVA, Proc GLM in SAS (9.1) to analyse effects relative to controls of BPA and EE2 on the different outcomes, followed by LSmeans test for differences between controls vs. the different treatment groups. Data were transformed when appropriate to meet requirements of homogeneity of variance among the treatment groups. Statistical significance (2 tailed) was set at *P* < 0.05.

## Supplementary Material

dvz012_Supplementary_Materials
