# Supplementary material for: Estrogen receptor 1 expression and methylation of Esr1 promoter in mouse fetal prostate mesenchymal cells induced by gestational exposure to bisphenol A or ethinylestradiol
Source: Environ Epigenet. 2019 Aug 22;5(3):dvz012. doi: 10.1093/eep/dvz012 (PMC6705189; doi:10.1093/eep/dvz012)

**SUPPLEMENTAL INFORMATION**

**Estrogen receptor 1 expression and methylation of *Esr1* promoter in mouse fetal prostate mesenchymal cells induced by gestational exposure to bisphenol A and ethinylestradiol**

Ramji K. Bhandari^†‡*^, Julia A. Taylor^†^, Jennifer Sommerfeld-Sager^†^, Donald E. Tillitt^‡^, William A. Ricke^, Frederick S. vom Saal^†^

^*^ Department of Biology, University of North Carolina at Greensboro, Greensboro, NC 27412

^†^Division of Biological Sciences, University of Missouri, Columbia, MO 65211

^‡^USGS Columbia Environmental Research Center, Columbia, MO 65201, U.S.A.

^Department of Urology, University of Wisconsin, Madison WI 53705

*Corresponding author

Email: [rkbhanda@uncg.edu](mailto:rkbhanda@uncg.edu)

**Table 1.** Primer sequences used for MSRE-qPCR and RT-qPCR assay. Internal primers did not contain any CpGs, so copy number generated by internal primers indicated total copy number of genomic DNA and was, therefore, used as an internal control to normalize DNA methylation in the sample.

| **Primers used for MSRE qPCR** | |  |
| --- | --- | --- |
| **Primer name** | **Forward** | **Reverse** |
| mESR1exon1A | ACTTGCGCTGCGCCTTCTCT | CTCTCCATGGGCATCTTGA |
| mESR1exon1C | ACTGCTGTCCCTCAGCAGAC | AAGGAAGGAATGTGCTCGAA |
| MSRE-mESR2 | CATCCGGGTCTGCAGTAGAG | CAGAGACTCACGGGCAGGT |
| Internal Control | AGTGTGATGTTCCCAGTAGTGC | TCTCCAACTTTACATACTCCTCTCC |
|  |  |  |
| **Primers used for RT-qPCR** | |  |
| **Gene** | **Forward** | **Reverse** |
| ESR1 | ATGAAAGGCGGCATACGGAAAG | CACCCATTTCATTTCGGCCTTC |
| ESR2 | CCAGACTGCAAGCCCAAATGT | AGAAGCGATGATTGGCAGTGG |
| GAPDH | ATGGTGAAGGTCGGTGTGAAC | GCCTTGACTGTGCCGTTGAAT |
| Dnmt1 | AAGAATGGTGTTGTCTACCGAC | CATCCAGGTTGCTCCCCTTG |
| Dnmt3a | GATGAGCCTGAGTATGAGGATGG | CAAGACACAATTCGGCCTGG |
| Dnmt3b | CTGTCCGAACCCGACATAGC | CCGGAAACTCCACAGGGTA |
| Cyp19a | ATGTTCTTGGAAATGCTGAACCC | AGGACCTGGTATTGAAGACGAG |

**Table 2.** Methylation (%) of all CpG sites in two restriction sites (*Hpa*II: C**CG**G or *Aci*I: C**CG**C) in the promoter of estrogen receptors. There were 2 CpG sites in *Esr1* promoter Exon 1A, 4 CpG sites in Exon 1C, and 4 CpG sites in *Esr2* promoter.

|  | **% methylation** | |
| --- | --- | --- |
| **Treatment** | ***ESR1 exon 1A*** | **SEM** |
| Control | 1.939943535 | 0.730741 |
| EE2 Low | 2.542308529 | 0.595909 |
| EE2 High | 2.160128598 | 0.125715 |
| BPA Low | 2.9553468 | 0.102678 |
| BPA High | 3.141610933 | 0.295872 |

|  | **% methylation** | |
| --- | --- | --- |
| **Treatment** | ***Esr1* exon 1C** | **SEM** |
| Control | 0.82242304 | 0.070643313 |
| EE2 0.04 | 1.198438072 | 0.33615452 |
| EE2 0.4 | 1.04898122 | 0.080355379 |
| BPA 5 | 1.106734752 | 0.135422629 |
| BPA 50 | 1.268350313 | 0.226068309 |

|  | **% methylation** | |
| --- | --- | --- |
| **Treatment** | ***Esr1* exon 1C** | **SEM** |
| Control | 0.82242304 | 0.070643313 |
| EE2 0.04 | 1.198438072 | 0.33615452 |
| EE2 0.4 | 1.04898122 | 0.080355379 |
| BPA 5 | 1.106734752 | 0.135422629 |
| BPA 50 | 1.268350313 | 0.226068309 |

|  |  |  |
| --- | --- | --- |
|  |  |  |
|  |  |  |
|  |  |  |
|  |  |  |
|  |  |  |
|  |  |  |

|  | **% methylation** | |
| --- | --- | --- |
| **Treatment** | ***Esr1* exon 1C** | **SEM** |
| Control | 0.82242304 | 0.070643313 |
| EE2 0.04 | 1.198438072 | 0.33615452 |
| EE2 0.4 | 1.04898122 | 0.080355379 |
| BPA 5 | 1.106734752 | 0.135422629 |
| BPA 50 | 1.268350313 | 0.226068309 |

**Fig. S1**

Genomic sequence used to generate primers for MSRE qPCR. >> marks indicate primer sequence and nucleotides bolded and underlined indicate restriction sites for methylation specific restriction enzymes (HpaII and AciI) and CpGs on the sequence are highlighted.

1. **Promoter: ESR1 Exon 1A**


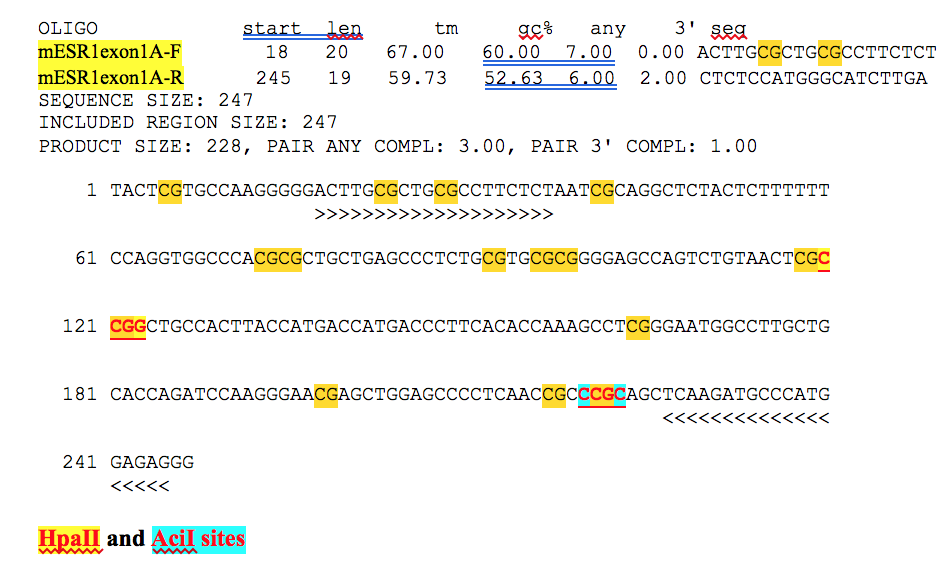


1. **Promoter: ESR1 Exon 1C**


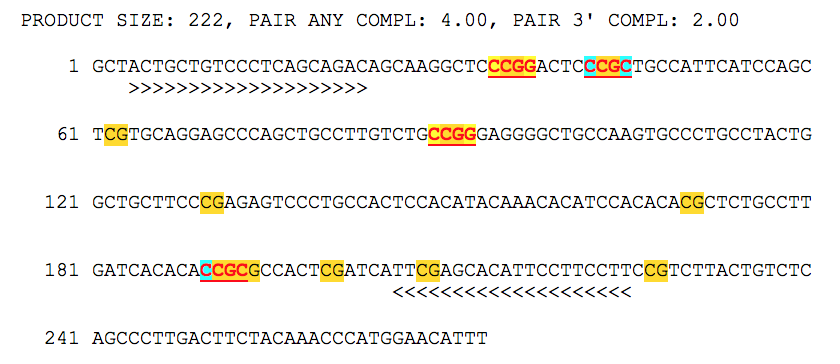


1. **ESR2 Promoter**


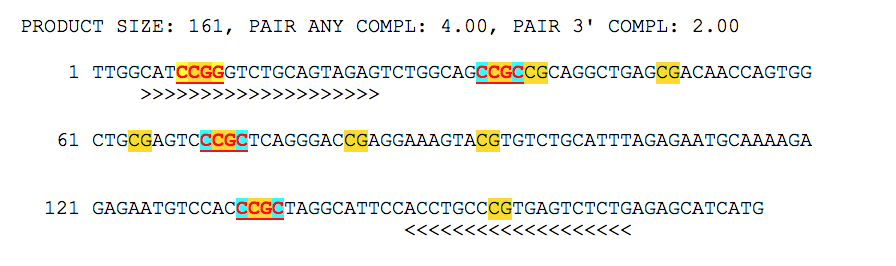


1. **Genomic region with no CpG site (internal control)**


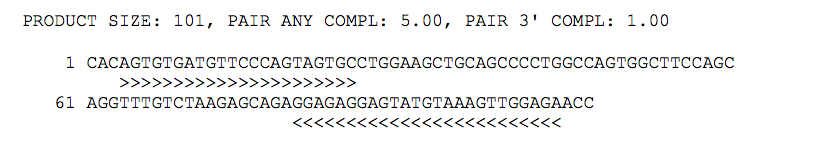

Supplement: dvz012_Supplementary_Materials [file dvz012_supplementary_materials.zip › dvz012_Supplementary_Materials.docx]
