# Supplementary material for: Estrogen receptor 1 expression and methylation of Esr1 promoter in mouse fetal prostate mesenchymal cells induced by gestational exposure to bisphenol A or ethinylestradiol
Source: Environ Epigenet. 2019 Aug 22;5(3):dvz012. doi: 10.1093/eep/dvz012 (PMC6705189; doi:10.1093/eep/dvz012)
Supplement: dvz012_Supplementary_Materials [file dvz012_supplementary_materials.zip › dvz012_Supplementary_Materials_Tables.docx]

**Table 1.** Primer sequences used for MSRE-qPCR and RT-qPCR assay. Internal primers did not contain any CpGs, so copy number generated by internal primers indicated total copy number of genomic DNA and was, therefore, used as an internal control to normalize DNA methylation in the sample.

| **Primers used for MSRE qPCR** | |  |
| --- | --- | --- |
| **Primer name** | **Forward** | **Reverse** |
| mESR1exon1A | ACTTGCGCTGCGCCTTCTCT | CTCTCCATGGGCATCTTGA |
| mESR1exon1C | ACTGCTGTCCCTCAGCAGAC | AAGGAAGGAATGTGCTCGAA |
| MSRE-mESR2 | CATCCGGGTCTGCAGTAGAG | CAGAGACTCACGGGCAGGT |
| Internal Control | AGTGTGATGTTCCCAGTAGTGC | TCTCCAACTTTACATACTCCTCTCC |
|  |  |  |
| **Primers used for RT-qPCR** | |  |
| **Gene** | **Forward** | **Reverse** |
| ESR1 | ATGAAAGGCGGCATACGGAAAG | CACCCATTTCATTTCGGCCTTC |
| ESR2 | CCAGACTGCAAGCCCAAATGT | AGAAGCGATGATTGGCAGTGG |
| GAPDH | ATGGTGAAGGTCGGTGTGAAC | GCCTTGACTGTGCCGTTGAAT |
| Dnmt1 | AAGAATGGTGTTGTCTACCGAC | CATCCAGGTTGCTCCCCTTG |
| Dnmt3a | GATGAGCCTGAGTATGAGGATGG | CAAGACACAATTCGGCCTGG |
| Dnmt3b | CTGTCCGAACCCGACATAGC | CCGGAAACTCCACAGGGTA |
| Cyp19a | ATGTTCTTGGAAATGCTGAACCC | AGGACCTGGTATTGAAGACGAG |

**Table 2.** Methylation (%) of all CpG sites in two restriction sites (*Hpa*II: C**CG**G or *Aci*I: C**CG**C) in the promoter of estrogen receptors. There were 2 CpG sites in *Esr1* promoter Exon 1A, 4 CpG sites in Exon 1C, and 4 CpG sites in *Esr2* promoter.

|  | **% methylation** | |
| --- | --- | --- |
| **Treatment** | ***ESR1 exon 1A*** | **SEM** |
| Control | 1.939943535 | 0.730741 |
| EE2 Low | 2.542308529 | 0.595909 |
| EE2 High | 2.160128598 | 0.125715 |
| BPA Low | 2.9553468 | 0.102678 |
| BPA High | 3.141610933 | 0.295872 |

|  | **% methylation** | |
| --- | --- | --- |
| **Treatment** | ***Esr1* exon 1C** | **SEM** |
| Control | 0.82242304 | 0.070643313 |
| EE2 0.04 | 1.198438072 | 0.33615452 |
| EE2 0.4 | 1.04898122 | 0.080355379 |
| BPA 5 | 1.106734752 | 0.135422629 |
| BPA 50 | 1.268350313 | 0.226068309 |

|  | **% methylation** | |
| --- | --- | --- |
| **Treatment** | ***Esr1* exon 1C** | **SEM** |
| Control | 0.82242304 | 0.070643313 |
| EE2 0.04 | 1.198438072 | 0.33615452 |
| EE2 0.4 | 1.04898122 | 0.080355379 |
| BPA 5 | 1.106734752 | 0.135422629 |
| BPA 50 | 1.268350313 | 0.226068309 |

|  |  |  |
| --- | --- | --- |
|  |  |  |
|  |  |  |
|  |  |  |
|  |  |  |
|  |  |  |
|  |  |  |

|  | **% methylation** | |
| --- | --- | --- |
| **Treatment** | ***Esr1* exon 1C** | **SEM** |
| Control | 0.82242304 | 0.070643313 |
| EE2 0.04 | 1.198438072 | 0.33615452 |
| EE2 0.4 | 1.04898122 | 0.080355379 |
| BPA 5 | 1.106734752 | 0.135422629 |
| BPA 50 | 1.268350313 | 0.226068309 |
